# A GDPR-Compliant Dynamic Consent Mobile Application for the Australasian Type-1 Diabetes Data Network

**DOI:** 10.3390/healthcare11040496

**Published:** 2023-02-08

**Authors:** Zhe Wang, Anthony Stell, Richard O. Sinnott, the ADDN Study Group

**Affiliations:** School of Computing and Information Systems, The University of Melbourne, Melbourne, VIC 3010, Australia

**Keywords:** GDPR, privacy, dynamic consent, mHealth, type-1 diabetes

## Abstract

Australia has a high prevalence of diabetes, with approximately 1.2 million Australians diagnosed with the disease. In 2012, the Australasian Diabetes Data Network (ADDN) was established with funding from the Juvenile Diabetes Research Foundation (JDRF). ADDN is a national diabetes registry which captures longitudinal information about patients with type-1 diabetes (T1D). Currently, the ADDN data are directly contributed from 42 paediatric and 17 adult diabetes centres across Australia and New Zealand, i.e., where the data are *pre-existing* in hospital systems and not manually entered into ADDN. The historical data in ADDN have been de-identified, and patients are initially afforded the opportunity to opt-out of being involved in the registry; however, moving forward, there is an increased demand from the clinical research community to utilise fully identifying data. This raises additional demands on the registry in terms of security, privacy, and the nature of patient consent. General Data Protection Regulation (GDPR) is an increasingly important mechanism allowing individuals to have the right to know about their health data and what those data are being used for. This paper presents a mobile application being designed to support the ADDN data collection and usage processes and aligning them with GDPR. The app utilises *Dynamic Consent*—an informed specific consent model, which allows participants to view and modify their research-driven consent decisions through an interactive interface. It focuses specifically on supporting dynamic opt-in consent to both the registry and to associated sub-projects requesting access to and use of the patient data for research purposes.

## 1. Introduction

In 2022, Optus, the second-largest telecommunication company in Australia, and Medibank, one of the major Australian private health insurance providers, suffered major data breaches [1,2,3]. These resulted in a range of sensitive data being released on the Internet, including Medicare numbers and health claims data. The increasingly frequent occurrence of such data breaches has placed millions of Australians’ personal data at risk. Through such breaches and many others, individuals have become more aware of what data are collected about them, where those data are located and how/why that information is being used.

Since 2020, the Australian government has been actively seeking public opinion on its current Australian privacy legislation—Privacy Act 1988 (The Act) [4,5,6], to bring it in line with international frameworks such as the European Union proposed General Data Protection Regulation (GDPR) [7]. Amongst other things, the Privacy Legislation Amendment 2022 aims to increase the penalties for serious privacy breaches [6,8]. GDPR recognises that companies and organisations across Australia and globally need to consider the protection and use of data over its lifetime. This is especially the case with health data. In contrast to the current Privacy Act, GDPR is a more advanced regulation that provides the definition of “personal data” instead of “personal information”, it broadens the scope of data under protection and gives individuals (“data subjects”) more control over their own data. It also outlines several legal bases that businesses and organisations are required to rely on to process personal data, among which the idea of *consent* is key. GDPR enumerates the conditions for legitimate consent to ensure the unambiguity, granularity and informativeness of consent and the autonomy of individuals when giving consent [7]. Individuals also have the right to withdraw their consent and hence data at any time with no recourse to themselves or the healthcare they may be receiving.

Personal data are ever-increasingly digitised and subsequently hosted by various organisations (GDPR “data controllers”). In the health context, this data can include personal data related to the individuals health, e.g., through Electronic Health Records (EHRs) [9]. Medical registries, biobanks, and clinical trials often collect and store health data to serve many diverse research purposes. For many research projects, a one-off opt-in or opt-out static consent model is the primary means of recording an individual’s agreement to engage in research and allow access to the use of their data. This is currently compliant with national legal and ethical requirements in Australia and New Zealand; however, the legal and ethical landscape is changing, especially with the rise and adoption of GDPR. The Australasian Diabetes Data Network (ADDN—www.addn.org.au) is one example of a one-off opt-out consent model. ADDN collates the health data of patients with diabetes from both paediatric and adult centres across Australasia to provide a national registry for approved researchers. Once an individual consents to be involved in ADDN, or more specifically, *they do not opt-out of being involved in the registry*, their data can be used for many diverse purposes (www.addn.org.au/publications accessed on 20 December 2022). For example, Couper et al. [10]. used patient Body Mass Index (BMI) data from ADDN to measure the impact on cardiovascular risk factors for youths with type-1 diabetes (T1D). There are also several international studies using ADDN data. For example, Bratina et al. [11] examined the prevalence of diabetic retinopathy among youths with type-1 diabetes (T1D) from 11 countries, where the Australian data were extracted from ADDN. The cross-border usage of ADDN data makes complying with international data sharing frameworks increasingly important. However, it is very likely that the patients themselves are unaware of these studies and how their data might be used. Furthermore, once they have not opted-out of the registry, their data are continually updated over time with no need to periodically check that they still consent to have their data stored in the registry.

The ADDN platform has been developed within the context of Australian legislation such as the Privacy Act 1988 (The Act) and its national equivalents (such as the NZ Privacy Act 2020) [12]. In [13], we explored the various technical, legal and ethical issues of ADDN that may be raised in the context of GDPR with a specific focus on fulfilling GDPR consent requirements, and the impact of GDPR on ADDN motivated us to design an application that resonates with the more advanced privacy regulation framework. This paper is an extension of [13]. Specifically, we present a prototype mobile Health (mHealth) application realising a GDPR dynamic consent model for ADDN that brings patients “into the loop” regarding data in the registry and how those data can be accessed and used. We present the conceptualisation and design of the new ADDN consent process, describing how dynamic GDPR-compliant consent is supported through the mHealth application that addresses the legal and ethical demands on the downstream use of the data by researchers, as well as the patients’ right to know what is happening with their data. We discuss the ramifications this has on the ADDN business as usual processes and how these need to be counter-balanced by the increasing demands of an individual’s right to know what is happening with their data.

## 2. Background

### 2.1. The ADDN Project

Australia’s prevalence of type-1 diabetes (T1D) is rated the sixth highest in the world, and the incidence in children aged 0–14 is the seventh highest. In September 2022, there were 134,735 people with type-1 diabetes registered with the National Diabetes Service Scheme (NDSS) [14]. The Australasian Diabetes Data Network (ADDN) registry is a clinical database comprising data from patients with T1D. It was established in 2012 and funded by the Juvenile Diabetes Research Foundation (JDRF) [15]. Currently, the data populated within the ADDN registry are uploaded from 42 paediatric and 17 adult diabetes centres across Australia and New Zealand. This comprises extensive data on over 20,000 patients with over 230,000 visits/treatments. ADDN has been established to enable the collection of health information from individual centre databases onto a single unified platform. This allows monitoring long-term outcomes for people with T1D; to facilitate T1D and related research studies, and ultimately to improve the clinical care of T1D patients across Australasia. The ADDN registry is developed, supported, and maintained by the Melbourne eResearch Group (MeG—www.eresearch.unimelb.edu.au) at the University of Melbourne.

Clapin et al. [16] describe Phase 1 (2012–2015) of the ADDN registry development. In this phase of ADDN, the focus was primarily on collecting and using data on paediatric patients coordinated with the Australasian Paediatric Endocrine Group (APEG [17]) and the Australian Diabetes Society (ADS [18]). ADDN Phase 1 realised a web-based registry with an agreed dataset and data dictionary. This included a core and extended (optional) set of data agreed on by all sites, clinicians, and researchers involved in APEG and ADS. This phase also included an overarching governance structure and guidelines for data access, and for the national reporting of diabetes outcomes in children and adolescents. A selected subset of diabetes centres made their data available through the ADDN registry in Phase 1. The ADDN project established operational practices for the cleaning and processing of the site data for incorporation into a unified, national registry.

**Figure 1:**
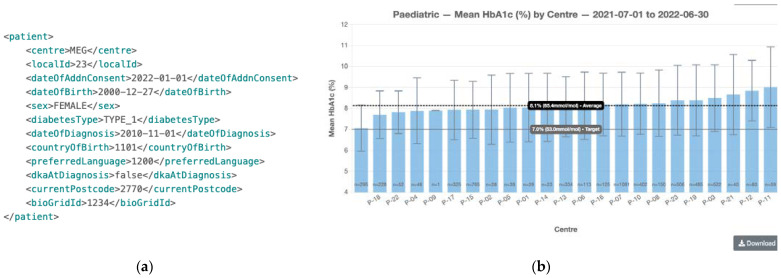
(**a**) A representative example of de-identified data based on a subset of the ADDN schema. (**b**) A screenshot of the HbA1c section of the benchmarking report. “P-“ indicates a paediatric centre.

Following the success of Phase 1, ADDN Phase 2 (2016–2020) included additional paediatric centres and a multitude of adult diabetes centres. These centres upload data to ADDN twice a year and receive a site-specific benchmarking report as a benefit (see Figure 1b). They are also informed of studies that might be occurring or propose their own studies using the aggregated T1D data. During this phase, ADDN data were made available for an extended set of research projects. These projects went through a light-touch ADDN-specific process for approval. However, communication of this to patients was not made, hence they were unaware of the projects that were proposed and/or taking place using their data.

The technical implementation and governance structure of the Phase 2 ADDN registry focused more on the security of data by de-identifying the data at the source and obtaining opt-out patient consent before populating to ADDN. Figure 1a gives a representative example of de-identified patient data based on the current ADDN schema, where the identifying data of patients have been removed and replaced by unique subject identifiers (USI) generated by the BioGrid data linkage platform (shown as *bioGridId*), together with a centre-specific *LocalId* and associated centre name. The date that the person decided not to opt-out of the ADDN registry is recorded as *dateOfAddnConsent*.

The current Phase 3 of ADDN (2021–2024) focuses on delivering better health outcomes for people with T1D and expanding the registry to many additional centres across Australia and New Zealand, as well as supporting international collaborations. Importantly, the project wishes to support deeper analytics and the linkage of ADDN data with data from external agencies such as the Australian Institute of Health and Welfare [19] national death index, the Australian medical benefits scheme [20] and the Australian pharmaceutical benefits scheme [21]. Such linkages necessitate the inclusion and use of more (fully) identifying data, i.e., fully identifying data are to be released from hospitals and included in the ADDN registry. The ethics approval for this has already been granted; however, this raises both technical, legal and broader ethical issues regarding data collection and usage that go beyond any given ethics review committee decision. As such, the impact of GDPR needs to be thoroughly considered, and especially what this means to the ADDN patients themselves.

### 2.2. GDPR Concepts

#### 2.2.1. Controller, Processor and Data Subjects

Articles 4.7 and 4.8 of GDPR [7] define *Data Controller* as an “entity that determines the ’why’ and the ’how’ of processing personal data”, whilst the definition of *Data Processor* is “the entity that actually performs the data processing on the controller’s behalf”. ADDN is a multi-party collaboration that has a complex chain of parties performing the duties of both the data controller and data processor. An ADDN governance team consisting of independent (external) investigators, ADDN investigators, JDRF representatives, and patient advisory group representatives determines the primary direction of operations of ADDN. The software developers at the Melbourne eResearch Group (MeG) are in charge of processing the data that comes from hospital systems and aligning it with ADDN governance structures. For example, site data can only be accessed by the sites directly, and only aggregated data can be released to researchers.

It is noted that GDPR defines the concept of a data subject as “any *living* individual whose personal data is collected, held or processed by a particular organisation.” (Article 4.1 [7]). Many of the patients in ADDN have since become deceased, so they are not data subjects in the GDPR sense. The personal data about living individuals (data subjects) fall into the scope of GDPR, and are thus under protection. GDPR then gives the definition of what personal data are.

#### 2.2.2. Personal Data, Pseudonymisation and Safeguards

According to Article 4.1 of GDPR [7], personal data are considered to be “any information concerning an identified or identifiable natural person”. Though there are no specific examples of personally identifiable information (PII) given by GDPR, we can refer to the instructive list of 18 identifiers (Box 1) introduced by the US Health Insurance Portability and Accountability Act (HIPAA) [22].

Box 1 **Personally identifiable information defined by HIPAA.**

(1) Name.
(2) Address (all geographic subdivisions smaller than state, including street address, city county, and zip code).
(3) All elements (except years) of dates related to an individual (including birthdate, admission date, discharge date, date of death, and exact age if over 89).
(4) Telephone numbers.
(5) Fax number.
(6) Email address.
(7) Social Security Number.
(8) Medical record number.
(9) Health plan beneficiary number.
(10) Account number.
(11) Certificate or license number.
(12) Vehicle identifiers and serial numbers, including license plate numbers.
(13) Device identifiers and serial numbers.
(14) Web URL.
(15) Internet Protocol (IP) address.
(16) Finger or voice print.
(17) Photographic image—photographic images are not limited to images of the face.
(18) Any other characteristic that could uniquely identify the individual.

Pseudonymisation is a de-identification procedure by which identifiable information is replaced by a unique key code; for example, by hashing or other suppression and obfuscation technologies [23]. It is recognised as a safeguard of personal data under GDPR, but pseudonymised data are still personal data if “it can be attributed to a natural person by the use of additional information” (Recital 26 [7]). GDPR describes appropriate safeguards as technical (e.g., pseudonymisation, encryption) or organizational measures (e.g., ethics committee responsible for governance) that should be used to minimize data leakage and privacy erosion (Recital 156 [7]). The data controller and processor need to align with such technical and organizational measures when processing personal data (Article 23 [7]) for public interest or for scientific research (Article 9.2 [7]) to be labelled as GDPR-compliant.

#### 2.2.3. Legal Bases

Data controllers are required to provide a legal basis to ensure the lawfulness of processing personal data under GDPR. In [13], we summarised the five legal bases researchers should rely on when involved in a medical research project, and more specifically, the six legal bases for processing sensitive personal data, i.e., “special categories of personal data”. For medical research where sensitive data are involved, such as ADDN, we rule out irrelevant legal bases, for example, the contractual service, legal obligation or vital interest are obviously not applicable. We concluded that informed consent would eventually become mandatory for processing sensitive health data without a public interest certificate or scientific research exemption in place with appropriate safeguards. The immediate challenge faced by ADDN is the current opt-out consent model is insufficient to comply with the conditions of GDPR consent.

Box 2 **Conditions of consent (Article 7).**

(1) ***Freely given***—the data subjects must not be cornered into agreeing, noting that the imbalance between the data subject and controller can often make unencumbered consent difficult, e.g., patients may feel obliged or have concerns that the treatments they receive may be inferior if they do not agree. Furthermore, each usage of personal data should be given separate consent.
(2) ***Specific***—the consent must be collected for certain agreed activities or purposes unless explicitly identified as “general” research.
(3) ***Informed***—the data subject must fully understand the consent before making the decision, including an understanding of data processing activities and their purpose and any associated risks or consequences.
(4) ***Unambiguous***—it should be immediately clear whether the data subject has consented. Consent under GDPR cannot be implied, and explicit opt-in consent is required.
(5) ***Withdrawal***—individuals can withdraw their consent at any time, and this withdrawal should be made as easy as obtaining the original consent.

#### 2.2.4. GDPR Consent Conditions

Informed opt-in consent is the legal basis upon which ADDN needs to rely upon moving forward to be aligned with GDPR. Consent is only valid when it meets the five conditions summarised in Box 2 above. There has been a long debate that the strict restrictions of GDPR legal bases and consent may be a “threat to hamper medical research” [24] since the purpose of medical research is often vague at the time of data collection and hence it would fail the “specific” conditions identified above. GDPR recognises “Consent to Certain Areas of Scientific Research” or “Broad Consent” for scientific use in Recital 33. That is, with appropriate safeguards, data subjects should be allowed to consent to certain areas of research using their data. However, Broad Consent is subject to a range of limitations, e.g., it cannot be used for primary research and is only limited to secondary research [25]. Additionally, the opponents of Broad Consent argue that it is by its nature uninformative [26], and the absence of a “broadness” standard may put personal data at risk of abuse, as the actual “broadness” can vary significantly depending on often subjective research-driven interpretations [27].

### 2.3. Dynamic Consent

#### 2.3.1. mHealth

Mobile health is the terminology used to define the usage of mobile communication devices including mobile phones, tablets and wearable devices, which pertain to the field of clinic research, health services and health monitoring [28]. The use of mHealth is growing rapidly as communication technologies improve. In 2022, Australia had the second-highest smartphone penetration rate in the world [29]. Diverse mobile health applications [30,31,32,33] have been developed based on the widespread adoption of smartphones.

Most mHealth applications are often concerned with removing temporal, geographic, and organizational barriers to clinical research and health services [34]. They enable patients, doctors, and researchers to easily access clinical data through mobile devices anytime and anywhere. Previous research projects have explored the benefits patients gained from mHealth applications by being active participants. For example, Qudah et al. [35] found mHealth would affect the relationship between patients and health providers positively and could ultimately improve health outcomes. Baysari et al. [36] identified that by enhancing the design of mHealth applications, “human factors” would facilitate patient-centred care coordination. Many mainstream apps from technology providers such as Apple have many health-related apps included directly onto the phone as part of the core operating system that track a range of health-related phenomenon, e.g., steps taken each day.

#### 2.3.2. eConsent and Dynamic Consent

Whilst most surveys focus on the user experience and efficacy of mHealth apps, only a few focus on their benefits from the perspective of personal data protection, privacy regulations and ethics. Schairer et al. [37] highlight the importance of electronic informed consent (eConsent) in mHealth applications. This mitigates the unforeseen privacy risks that patient data may be exposed to when existing in complex mHealth ecosystems by handling their data in a transparent manner.

The benefits of eConsent can only be realized with proper patient-focused co-design processes, where patients are at the heart of the technology design and evolution. Different models of consent have been put forward to be in line with changing regulations, evolving ethical requirements and diverse research needs, but these are rarely exposed directly to the needs, demands and understanding of patients themselves. Wiertz et al. [27] focused on different models of consent and their ethical concerns. They considered Tiered Consent, Meta Consent involving Broad Consent, and specific Dynamic Consent through interactive and personalised interfaces. Dynamic Consent was first implemented in the Ensuring Consent and Revocation (EnCoRe) project [38]. Dynamic Consent was formally defined by Kaye et al. in [32]. It was initially designed for biobanks where personal (physical) data were often reused by many research projects and researchers. However, Broad Consent is often not immediately informative in this context to patients, since the complexities of genomic data processing and the ramifications this might have to patients are often complex. With Dynamic Consent, research participants are allowed to dynamically revise their granular consent options to new or existing data access requests over time and constantly communicate with research teams through interactive software interfaces [39]. The nature of Dynamic Consent enables participant-centred research and resonates with the GDPR consent requirements focused on inclusive design, i.e., instead of being passive research subjects in one-off static consent settings, data subjects are actively involved in the consent process, and can consent or withdraw their consent in a free, unencumbered, and real-time manner to many diverse research projects that the patients are made aware of through a mobile app.

#### 2.3.3. Implementation of Dynamic Consent

Over the past decade, Dynamic Consent has been implemented in various biobank and clinical data network projects. The Cooperative Health Research in South Tyrol (CHRIS) explored Dynamic Consent [40,41]. They recruited over 13,000 participants who agreed to the storage of their biological (physical) data in a biobank. Michigan’s BioTrust for Health [42] piloted a dynamic consent web solution with 187 testers and found a strong preference for active engagement in biobank-related research. Dynamic Consent has also been used in disease registries such as the Rare UK Diseases of bone, joints and blood vessels study (RUDY) [43,44]. RUDY provides a rare disease research network that aggregates clinical events provided by patients with a range of rare diseases. RUDY uses dynamic consent as part of its broader software solution. Both RUDY and CHRIS adopted dynamic consent at their inception. There has been no system that focused on embedding a dynamic consent process into an existing complex national infrastructure where the consent model was based on a less dynamic and non-granular, one time opt-out consent model.

From a GDPR consent perspective, Dynamic Consent helps to fulfil several key consent requirements. For example, CHRIS offers no financial compensation for participation, thereby ensuring that consent is freely given. RUDY allows participants to select their involvement in different sub-studies and allows them to change their consent at any time. Educational videos and FAQ pages are provided in the BioTrust project. These aim to support informed consent. An explicit “Yes” option is compulsory for participation in CHRIS, i.e., it is based on an unambiguous opt-in decision made by data subjects. However, the withdrawal conditions associated with GDPR consent are often neglected when implementing Dynamic Consent. Taking the CHRIS project as an example, whilst most choices are editable in the online platform, a complete withdrawal from the study is only available by contacting the study centre. This violates the GDPR “withdraw” conditions, as the withdrawal is made more difficult than the original giving consent, which only requires a click of “yes” on the platform.

Additionally, most Dynamic Consent implementations require identifying data for further patient contact. For example, the RUDY and CHRIS projects send letters and/or email notifications to participants to advise them of new requests they may wish to consent to. Dynamic Consent in these projects is built upon a secure web interface. However, without identifying data such as phone numbers or email, notifications cannot be sent to participants. The BioTrust project found that participants had concerns about the identity verification process and would prefer their samples to be de-identified. Mobile app-based push notifications can provide a potential solution to this. That is, a message can be sent by a server such as the ADDN registry to an mHealth app and hence to a participant without the details of the individual patient details leaving the ADDN registry, so long as they have the app installed on their mobile device and suitable activation codes are used to activate and target the mobile app to the specific patient on the registry. Such de-identification is an essential safeguard recognised by GDPR to lower the risk of potential data leakage and privacy erosion.

#### 2.3.4. Challenges of Dynamic Consent

There are various criticisms of Dynamic Consent. Most notably, significant resources and cost are required for its implementation [27], this challenge is exacerbated when building a dynamic consent process for large, complex (national) systems. A de-coupled, lightweight, and reusable application would be preferred. Dynamic Consent can also be a burden for research and potentially lower the research value due to lower patient opt-in rates [45]. It may also cause “consent fatigue”, as participants may stop paying attention and give “superficial consent” if they receive too many consent requests [27]. Data controllers need to consider such challenges when choosing a consent model for their research project. We discuss this in the context of ADDN in Section 3.5.

## 3. Dynamic Consent Implementation in ADDN

Hospitals have many thousands of patients, and patient data are regularly collected as part of routine health care. The diverse downstream use of these data within projects such as ADDN and the associated ADDN research sub-projects often happens without a patient’s full awareness and/or explicit opt-in consent for given studies. As noted, placing the patient into the heart of the data use process is essential to meet the core GDPR criteria. In this section, we introduce a prototype mobile application: *ADDN Consent*, that realizes a Dynamic Consent workflow for the ADDN project. The goal is to factor in GDPR consent demands by enabling patients to actively engage and support downstream ADDN research in a patient-driven, yet completely de-identified manner.

### 3.1. Recruitment and Onboarding

**Figure 2:**
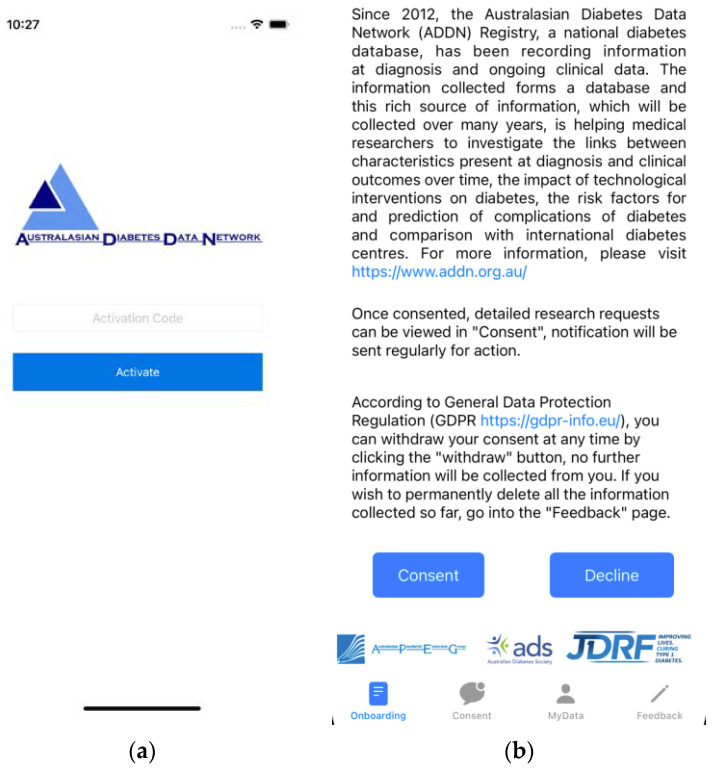
Screenshots of the ADDN Consent application. (**a**) Activation through an ADDN registry generated activation code. (**b**) Description of the ADDN project and basic onboarding documentation.

Currently within ADDN, participants (both existing and new) who attend clinics are provided the opportunity to opt-out of the ADDN registry by their clinician and/or nurses who treat them. Once they have gone through this process, this status is flagged in the local hospital system as *dateOfAddnConsent* in Figure 1a, and their data may be released to ADDN (or not). This process happens just once, and if they do not opt-out, their data are released and integrated into the ADDN registry twice per year thereafter. The downstream use of these data is not known to the patient. To support Dynamic Consent (opt-in), a new process requires hospital staff to generate an activation code on the ADDN registry and advise patients to install the *ADDN Consent* app. This can be done prior to the patient arrival at the clinic or during their clinic attendance. At this stage, patients are fully identified in the clinics. Clinicians will explain the ADDN project and the role of the *ADDN Consent* app for future downstream engagement and interactions with patients in the use of their data. Once the patient agrees to use the *ADDN Consent* app, an activation code is generated and input. This code is unique to the patient (see Figure 2a) and generated on the ADDN registry. This code means nothing outside the context of the ADDN registry, since the activation code itself is salted and hashed.

Once the app has been activated, patients are shown an onboarding page (Figure 2b) with terms and conditions and basic information regarding the ADDN project. At this point, they may opt-in consent to be involved in the registry. If they choose to withdraw from the registry, their data are flagged on the ADDN registry as “to be removed”. The patients may also choose to withdraw from the registry at any time. Once set, the data will be securely removed and will not be included in any future data uploads or subsequently released to any researchers.

After clicking the “Consent” button (Figure 2b), the patient has finished the onboarding step. At this point, they may receive notifications of future studies from researchers that wish to access and use their data. Patients may consent or decline to be involved in these future studies on a case-by-case basis. It is important to note that the ADDN Study Group are still the first line of assessment of any given research sub-project proposal. They may reject a request for scientific or other reasons, before a patient is ever notified.

### 3.2. Sub-Project Consent Process

**Figure 3:**
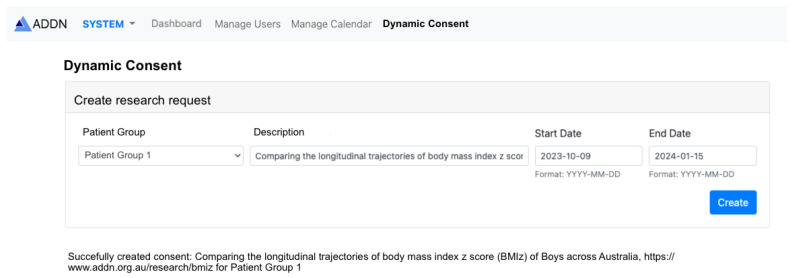
Screenshots of the ADDN registry: creating a research request.

The ADDN Study Group regularly receives requests from researchers that wish to access and use the ADDN data for research purposes. These requests are assessed and accepted or rejected depending on a range of factors, e.g., they might be rejected if there are insufficient data on the topic of research interest, or they may be rejected if there is a perceived risk to the patients that are involved, or indeed if the science is flawed or repeats existing work. Assuming that a research request is approved by the ADDN Study Group, the ADDN registry includes the details of the study. Figure 3 shows an example of a research request focused on “*Comparing the longitudinal trajectories of body mass index z score (BMIz) of boys across Australia, https://www.addn.org.au/research/bmiz*” created by an authorised administrator in the ADDN registry. All individuals in the ADDN registry who in principle meet the criteria for the study need to be notified through the mobile application. In this case, the study focuses explicitly on Australian, male patients 0–12 years of age. The server generates notifications that are sent to those patients who have the app installed and who meet the criteria for the study. A timeline for consenting/declining to be involved as well as a link for the study details are provided. This includes the layperson’s patient information sheets for the study and the more detailed scientific background to the study that is submitted to the ADDN Study Group.

**Figure 4:**
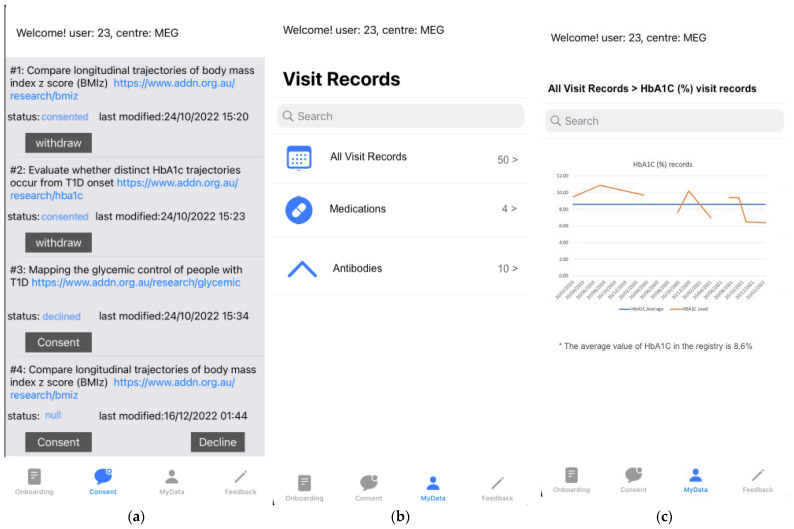
Screenshots of the ADDN Consent application. (**a**) Dynamic consent tasks. (**b**) Patient view of their data. (**c**) Patient view of their HbA1C data in visit records.

At this point, as shown in Figure 4a, patients (or in this case, the parents of the children who have installed the app) can review the details of the research project. The initial status of a sub-project consent task is “Null” (task #4 in Figure 4a). Patients can subsequently change the consent status to consent, decline or withdraw by clicking the buttons Consent, Decline or Withdraw, respectively, where:

- ***Consent*:** shows when the current status of the patient consent is not “Consented”. By clicking this button, patients are requested to agree to the terms and conditions of the study, and hence they agree to opt-in to the research project. It is noted that at present, the *ADDN Consent* app is purely focused on obtaining patient consent for studies using the ADDN registry data and not for the collection of additional data from patients.
- ***Decline***: this button shows when the status of the consent is not yet set (“Null”). By clicking this button, patients are able to freely decide to reject the consent task and thereby indicate that they do not wish their data to be used in the research.
- ***Withdraw***: this button shows when the consent status is “Consented”. By clicking this button, patients can withdraw their consent and, thereafter, their data will be removed and no longer used in the given research project.

Table 1 summarises the four consent statuses and the actions that the patient can perform depending on the consent status.

It is noted that the studies identified thus far do not request further data from the patients themselves. Rather they focus on whether the patients wish to consent to the access and use of their data for a specific research project.

### 3.3. Patient View of Their Data

Patients have the option of viewing their own data that exist on the ADDN registry ((Figure 4b,c) MyData). This includes longitudinal data such as their historic visit (e.g., HbA1C records) and medication records, as well as how they compare with other patients on the registry, noting that the other patient data are anonymised. That is all ADDN registry patients are benchmarked, and only the patient will be aware of their own data and how they compare to the other patients.

**Figure 5:**
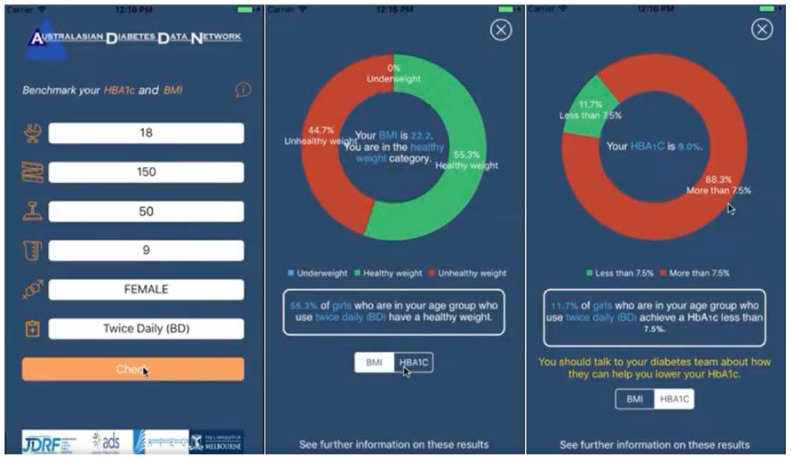
ADDN mobile app for comparison of patient data with registry data.

This is aligned with the more general ADDN mobile app that was developed and released to the Apple App Store and Google Play as shown in Figure 5. Here, individuals include their age, height, weight, HBA1c, gender and treatment information. This then compares them with other patients in the registry in their demographic and treatment profile.

### 3.4. Incorporating Dynamic Consent Data into the ADDN Data Load

**Figure 6:**
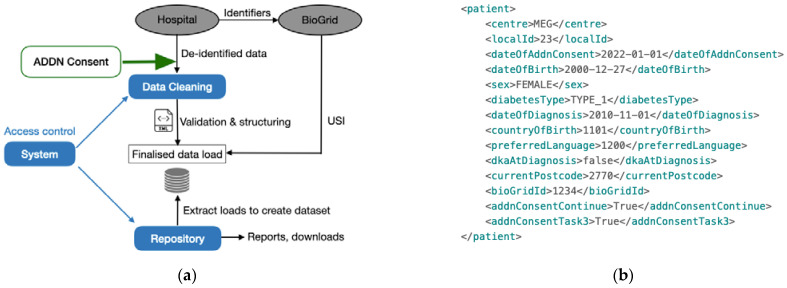
(**a**) Architecture of ADDN registry and data pipelines [12]. (**b**) An example of patient data with a merged opt-in consent result.

As mentioned, participating ADDN centres upload (XML) exports of their data (Figure 1a) to the ADDN registry twice a year. This undergoes extraction, transformation, and loading (ETL) functionalities, as discussed in [11]. As shown in Figure 6a, currently data are validated and structured before being uploaded to the database for use by clinicians and researchers. The cleaned and finalised data set is then, subject to the ADDN Study Group, made available for research. Patients may confirm (opt-in) to continue to be involved in ADDN by using the *ADDN Consent* app. As shown in Figure 6a, the *ADDN Consent* app serves as an additional data source to be merged with the main ADDN data integration task. For example, as the merged data shows in Figure 6b, by adding new opt-in consent fields, there will be three (types) of consent fields:

- *<addnConsentContinue>:* This indicates the patient has activated the app and made an opt-in decision of whether they still want to be in the registry or not, by clicking buttons on the “Onboarding” page (Figure 2b);
- *<addnConsentTaskX*>: This indicates the patient has made a decision regarding whether they wish to be involved in Research task X (e.g., <addnConsentTask3> for research task #3) by clicking buttons on the “Consent” page (Figure 4a);
- *<dateOfAddnConsent>:* This is the historical opt-out data collected by ADDN. It is noted that eventually it is expected that this field will be replaced by the above opt-in consent for all patients to comply with GDPR.

When creating a research dataset for a given study, an inclusion criterion specific to the study needs to be defined and set to True using the ADDN Consent mobile app, i.e., both opt-in decisions *<addnConsentContinue>* and *<addnConsentTaskX*> need to be True. In this way, when creating different datasets for each research task, the data without explicit opt-in consent for that research task will be excluded.

### 3.5. ADDN Consent Workflow

**Figure 7:**
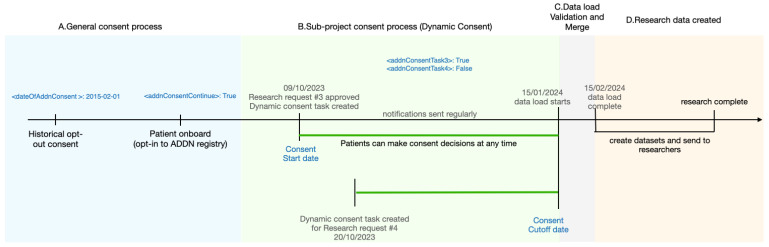
The complete ADDN Consent workflow.

The ADDN Consent workflow aims to facilitate research and be compliant with GDPR. Figure 7 gives an overview of the complete ADDN Consent workflow with a mock research request.

A. ***General consent process:*** as described in Section 3.1, patients who did not opt-out will be instructed by clinicians when visiting the clinic to activate the mobile consent app and finish the onboarding process. In this stage, patients make their general consent decisions where they can choose to opt-out/decline/withdraw and be removed from the ADDN registry, whereupon they will not receive any further notifications.
B. ***Sub-project consent process:*** as described in Section 3.2, in this example, two research requests are approved by the ADDN Study Group on 09/10/2023 and 20/10/2023. The ADDN registry administrator creates a view of the data for the patients that match the study criteria, e.g., 0–12 year-old boys, and subsequently creates a dynamic consent task #3 and #4 for these research projects. A notification is then sent to the target patient group’s mobile app. From this point, notifications are pushed to patients’ phones periodically until they make a decision (consent, decline, withdraw). Patients are allowed to modify their decision any time before the cut-off date—given here as 15/01/2024, when the next data load begins, and the centres start to send the updated data to ADDN.
C ***Data load validation and merge***: Patients’ decisions will be incorporated into the data load as inclusion criteria as described in Section 3.4.
D. ***Research data created***: for patients who have not opted-in, their data will be excluded from given research requests, and hence it will not be sent to researchers. In this example, the patients can choose to be involved in #3, but not for #4, i.e., the related data will be excluded when creating a dataset for research task #4.

It is noted that multiple research requests can be processed in parallel following the workflow indicated in tasks #3 and #4 in Figure 7.

### 3.6. Discussion

In light of the dynamic consent challenges summarised in Section 2.3.4, we consider if Dynamic Consent would be a suitable consent model for GDPR. Considering the cost and effort of building an application, a de-coupled consent mobile application that serves as an additional data source is more economic and efficient compared to embedding it directly into the research platform on a per study basis. Compared to existing realisations of Dynamic Consent as discussed in Section 2.3.3, ADDN Consent is a lightweight mobile application that demonstrates a technical realisation implementing Dynamic Consent in a large, national data-sharing framework.

From the perspective of research outcomes—different from most biobanks, where samples are collected from a large population—the data contributors of the ADDN registry are patients who are or might be experiencing diabetes, and hence they are more likely to become active partners, i.e., they have a vested interest in T1D and improving health care and outcomes for patients. The workflow described in Section 3.5 enables multiple research consent requests to be processed simultaneously. Additionally, the altruistic benefits of sharing data may prompt patients to be more actively engaged in the research [46].

When considering GDPR consent conditions, as described in Section 3.2, patients are able to view the details of particular research projects as well as the core ADDN data that are collected about them on the registry. Researchers are obliged to prepare additional information for non-experts, so patients are fully aware of what the research is about and the consequences and outcomes to them, hence the goal to comply with the GDPR “informed” and “specific” bases is met. Patients who to agree to a given study are still able to use the mobile app to view any current or past research projects of ADDN, i.e., their decision will not impact on their healthcare, thus meeting the GDPR “freely given” condition. Additionally, compared with in-person paper-based consent, the mobile consent app enables patients to make decisions anywhere, hence they have plenty of time to consider and consult families or friends, which can enhance their comprehension and autonomy compared to the decision made in a hospital setting, where they may feel obliged to say yes. An “unambiguous” opt-in consent provides an inclusion criterion of patient data to be included in the registry for future research projects. Compared to existing products as discussed in Section 2.3.3, where the “withdrawal” condition is easily neglected, we emphasise the simplicity of withdrawal in the *ADDN Consent* app. Patients can withdraw their consent at any time by simply clicking a button, and the withdrawal action is as easy as the consent action.

Furthermore, and perhaps most importantly from an ethical perspective, opt-out consent faces ethical challenges since patients are unaware of the downstream use of their data. *ADDN Consent* ensures passive research participants become active decision-makers. Participants will know exactly what data are collected about them, where it is located and how/why it is being used, and can make informed and granular consent decisions on the use of their data.

## 4. Conclusions

Our previous paper [13] explored the impact of GDPR on the ADDN platform with a focus on the various technical, legal and ethical problems associated with a national registry storing a large amount of health data originating from many health systems. As an extension, this paper focuses on the challenges of improving the consent process to better meet the conditions of GDPR for ADDN, i.e., moving from a one time, opt-out model to an explicit patient-centred opt-in model. We presented a prototype mobile app utilising a GDPR dynamic consent model: *ADDN Consent* and compared it to existing implementations of dynamic consent. We identified how the app provides a specific mapping of the GDPR conditions as part of an existing large data-sharing framework with associated data collection and patient interaction processes. We discussed the major challenges of the Dynamic Consent model and its suitability in ADDN, and how it can be incorporated into the current routine healthcare processes. A complete workflow of ADDN with a dynamic opt-in consent process along with the potential ramifications was presented. Currently, the prototype consent mobile app is built in the context of the ADDN project, but the application is de-coupled from the project and can be adopted in other clinical or medical research contexts for GDPR compliance with project-specific modifications.

The future work of *ADDN Consent* is to recruit participants to test the mobile application. Specifically, we plan to test the mobile application in a whole data load workflow to serve as an additional consent data source for the existing routine healthcare processes. We will also collect patient feedback and improve the solution. We also propose to build a real-time statistics dashboard recording the consent process to help save researcher time and facilitate the consent process, e.g., identify which research projects obtain consent and those that do not.

Moving forward, we will explore more GDPR-related functionalities in the app, including the right to be forgotten and the right to data portability, to better empower participants.

## Figures and Tables

**Table 1:** Consent status and associated actions.

Status/Action	Consent	Withdraw	Decline
**Consented**: the patient has opted-in to the study and their data can be used in the study	N	Y	N
**Withdrawn**: the patient has withdrawn their consent from an ongoing study, they wish their data to be removed and they no longer wish to be notified about the study	Y	N	N
**Declined**: the patient does not wish to be involved in a given (proposed) study and no longer wishes to be notified	Y	N	N
**Null**: no action yet, notifications can still be received	Y	N	Y

“Y” indicates an action that can be performed in a certain status (button shown), “N” indicates an action that cannot be performed in a certain status (button not shown).

## Data Availability

Not applicable.
